# Advancements in research on fluid overload in preterm infants—a narrative review

**DOI:** 10.3389/fped.2025.1691874

**Published:** 2026-01-07

**Authors:** Xinyi Liu, Shuyue Deng, Siyu Chen, Linxiao Wan, Wenbin Dong, Lan Kang

**Affiliations:** 1 Department of Neonatology, Children’s Medical Center, The Affiliated Hospital of Southwest Medical University, Luzhou, Sichuan, China; 2 Department of Perinatology, The Affiliated Hospital of Southwest Medical University, Luzhou, Sichuan, China; 3 Sichuan Clinical Research Center for Birth Defects, Luzhou, Sichuan, China

**Keywords:** adverse outcome, fluid overload, management of fluids, mechanisms, preterm infant

## Abstract

**Objective:**

Fluid overload (FO) is a prevalent clinical challenge in preterm infants, contributing to multiorgan dysfunction and adverse outcomes. This review synthesizes the pathophysiology, clinical implications, and management strategies of FO to advance fluid management in preterm neonates.

**Methods:**

We reviewed literature to define FO criteria, delineate its mechanisms (e.g., renal immaturity, endothelial glycocalyx impairment), and analyze associations with systemic complications. Current monitoring technologies and therapeutic interventions were evaluated. As a narrative review, literature identification and data extraction were conducted based on the research question and inclusion criteria without adhering to formal systematic review guidelines. All original studies cited in this review reported obtaining approval from institutional ethics committees and informed consent from parents or guardians. As a secondary analysis of published literature, this study does not involve new patient data and therefore required no additional ethical approval.

**Results:**

FO pathogenesis involves underdeveloped renal function, compromised skin barriers, glycocalyx damage, and hypoalbuminemia. FO can lead to multisystem adverse outcomes. Noninvasive monitoring—echocardiography, bioelectrical impedance analysis (BIA), and lung ultrasound—demonstrates high clinical utility. Effective management includes strict fluid restriction, diuretic, and albumin infusion.

**Conclusion:**

Optimizing FO management requires multimodal monitoring and individualized fluid regimens. Future research should prioritize refining assessment standards and developing targeted interventions to improve neonatal outcomes.

## 1 Introduction

According to World Health Organization (WHO) statistics, approximately 15 million preterm infants are born globally each year, accounting for over 10% of all births. The most recent global survey indicates that there were 13.4 million preterm births in 2020, representing 9.9% of all newborns. The global number of preterm births and their corresponding proportion have not shown a significant decline over the past decade (1, 2). Globally, among newborns from 1990 to 2022, the age standardized incidence rate (ASIR) of preterm birth increased by an average of 0.25% per year in high socio-demographic index (SDI) countries—such as Greece, Bahrain, Japan, the United Kingdom, and the United States. In contrast, the age-standardized mortality rate (ASMR) for preterm infants increased by an average of 2.09% per year in low-income countries, particularly in sub-Saharan Africa (3). Approximately 15% of these preterm infants were born at a gestational age under 32 weeks, necessitating more advanced neonatal care techniques. Appropriate fluid and electrolyte supplementation is critical in the management of preterm infants. Both Fluid Overload (FO) or inadequate administration and electrolyte disturbances can lead to severe complications, including brain injury, necrotizing enterocolitis, bronchopulmonary dysplasia, and hemodynamically significant patent ductus arteriosus (4, 5).

Fluid intake in preterm infants encompasses oral feeding, parenteral nutrition supplementation, and intravenous medication administration, while fluid output includes insensible water loss, urine, and fecal excretion, with the majority of insensible water loss occurring through the skin. The balance between fluid intake and output is critically important for maintaining homeostasis in preterm neonates. Challenges in fluid management arise due to immature renal function, underdeveloped skin barrier, and physiological postnatal fluid reduction in this population. This article provides a comprehensive review of the mechanisms underlying FO in preterm infants and examines the diseases associated with fluid overload in this vulnerable group.

## 2 Overview of FO

Fluid Overload (FO) is defined as a pathological accumulation of fluid resulting in an imbalance where total body water exceeds physiological requirements. This condition is particularly prevalent among preterm infants, especially those receiving intravenous fluids or parenteral nutrition. Due to the immaturity and rapid postnatal changes across organ systems in preterm infants, a universally accepted quantitative definition of FO in this population remains elusive. The term “fluid overload” was coined by Goldstein et al. in 2001 following their investigation of complications in critically ill pediatric patients undergoing continuous renal replacement therapy (CRRT) (6). They defined FO as a percentage fluid accumulation exceeding 10%, calculated as the difference between total fluid intake and output divided by admission body weight. The Society of Critical Care Medicine in the United States (SCCM) subsequently established >10% fluid overload as a critical threshold in managing septic shock in neonates and children (7). Existing literature primarily utilizes two metrics for defining FO: one based on fluid input and output measurements, and the other based on changes in body weight (8, 9). For instance, a fluid overload greater than 10% is considered significant, as calculated by the following formula:

Based on fluid balance: Cumulative fluid balance (%) = [(Cumulative fluid intake (mL) − Fluid output (mL))/Body weight (kg)] × 100%.
Based on body weight changes: Cumulative body weight change (%) = [(Current body weight (kg) − Estimated dry weight (kg))/Birth weight (kg)] × 100%.

Both formulas exhibit inherent limitations. The cumulative balance method calculates the difference between fluid intake and output, offering a unique advantage in managing critically ill preterm infants during the acute phase by allowing dynamic monitoring without needing to move the patient. However, fluid balance calculations often fail to account for insensible fluid losses, which can constitute a significant portion of total output in preterm infants. These losses are influenced by factors such as exposed body surface area, incubator temperature and humidity, ambient air conditions, and ventilator use. Additionally, estimating the volume of urine and stool can be challenging (10). In contrast, changes in body weight serve as a direct indicator of total body fluid volume and are theoretically more accurate. Yet, their use is limited by the difficulty of weighing critically ill preterm infants, and small measurement errors can be amplified due to their low body weight, leading to significant deviations. Therefore, the choice between the two formulas depends on clinical feasibility: the first formula is primarily used for hemodynamically unstable preterm infants who cannot be weighed, while the second is applied to more stable preterm infants for whom accurate weight measurements are available. Relevant studies have demonstrated that both weight-based and fluid balance-based approaches possess comparable predictive value for assessing FO (11). A summary of the relevant FO thresholds and their predictive values is presented in Table 1.

**Table 1:** Diagnostic thresholds of FO and predictive values.

Reference	Study population and design	Calculation for FO	Risk threshold	Predicted value
Li et al. (34).	ELBWI (*n* = 157) single-center retrospective case-control study	(Fluid intake-fluid output) (L)*100%/daily weight (kg)	36.2% (for 7 days total)	A 7-day cumulative FO is an independent risk factor for the development of BPD in ELBWI. A threshold of 36.2% cumulative FO over 7 days is the best predictor for the development of BPD in VLBWI.
Matsushita et al. (38).	ELBWI (*n* = 2,019) single-center retrospective cohort study	[Cumulative fluid input (mL) − output (mL)] × 100%/birth weight (kg)	Mild FO group (0∼10%) moderate FO group (10%∼15%) severe FO group (over 15%)	The mortality rate in the severe FO group (72.2%) was higher than that in the fluid negative balance group (28.6%), the mild group (30.4%), and the moderate group (23.4%) (*P* = 0.002).
Soullane et al. (35).	Preterm infants <29 weeks (*n* = 31) single-center retrospective cohort study	Fluid intake (dL/day) − fluid output (dL/day)/birth weight (kg)	101 mL/kg (on day 5)	Cumulative fluid balance on day 5 (below 1.01 dL/kg = 101 mL/kg) had high sensitivity (86%) to predict death/BPD.
Selewski et al. (39).	Near-term/term infants (*n* = 58) multicenter prospective cohort study	[1 − (weight at day 3/birthweight)(kg)] × 100%	8.2% (for 3 days total)	In infants with AKI, the percentage change in body weight on the third day was a median of 8.2% (IQR: 4.4 to 21.6). Those without AKI had a median weight change of −4% (IQR: −6.5 to 0.0) (*p* < 0.001).
Zozaya et al. (49).	Preterm infants <29 weeks (*n* = 9,275) multicenter retrospective cohort study	[Weight at day 3(kg) −birthweight (kg)] × 100%/birthweight	−13% to −9%	The change in body weight during the first 3 days after birth exhibited a “U”-shaped relationship with severe neurological injury in the fitted quadratic curve, where the lowest risk of neurological injury corresponded to a weight change range of −13% to −9%.
Valentine et al. (50).	Preterm infants 24–27 weeks gestational age (*n* = 941) a multicenter randomized, double-blinded, controlled clinical trial	[Weight at day 7(kg) −birthweight(kg)]/birthweight × 100%	5%–15%	There is a potential window of optimal physiologic weight loss of 5%–15% in the ﬁrst week after birth associated with decreased odds of NEC

ELBWI, very low-birth-weight infants; IQR, interquartile range.

## 3 Mechanisms underlying the occurrence of FO in preterm infants

### 3.1 Insufficient capacity for body fluid regulation

The stratum corneum of preterm infants is extremely thin at birth, measuring only a few micrometers. Due to defects in dermal structural proteins, their skin is highly susceptible to injury or tearing. Since the skin's barrier function relies almost entirely on the integrity of the stratum corneum (12, 13), preterm infants experience a relatively high proportion of transepidermal water loss. Moreover, there is a negative correlation between the degree of transepidermal water loss and gestational age at birth.

Additionally, The immature kidney function in preterm infants contributes to fluid imbalance. Fetal kidney maturation occurs in three stages, with the most active period being the final stage—metanephric development. Over 60% of the kidney units in a fetus are formed during the last three months of pregnancy, with development continuing until about 34–36 weeks of gestation (14, 15). Preterm infants are born before this critical point in kidney development, resulting in a significantly lower number of kidney units compared to full-term infants. Sutherland MR and colleagues conducted kidney autopsies on 28 preterm infants (gestational age 27.9 ± 0.7 weeks, postnatal survival 18.3 ± 3.4 days) and compared them with kidneys from stillborn fetuses of matched gestational age. The autopsy results showed that preterm infants had not only a lower proportion of immature stage V (vesicle stage) glomeruli compared to stillborns of the same gestational age but also a higher proportion of mature stage II/III glomeruli. This developmental difference was most pronounced in extremely preterm infants. These findings suggest that preterm birth can induce accelerated kidney maturation, and this effect is positively correlated with gestational age (16). The kidneys of preterm infants exhibit an increased cross-sectional area of renal corpuscles, with about 13% of glomeruli showing abnormal morphology, which can lead to hyperfiltration. Aquaporins (AQPs) are a group of specific channel proteins embedded in cell membranes, primarily responsible for the efficient and selective transport of water molecules. Thirteen mammalian AQP subtypes have been identified, nine of which are present in the human kidney. Among them, AQP2 is the main renal target of antidiuretic hormone and plays a crucial role in urine concentration (17, 18). Preterm infants have reduced levels of aquaporin receptors in their renal tubules, resulting in poor urine concentrating ability. Related studies have found that the urinary excretion of AQP2 in preterm infants 2–3 weeks after birth increases with higher gestational age at birth (17). Approximately 80% of sodium is reabsorbed in the proximal tubules. In preterm infants, the epithelial cells of the proximal tubules are not fully developed, resulting in reduced activity of the sodium pump (Na⁺/K⁺-ATPase) and a significantly decreased ability to reabsorb sodium. Additionally, the immature development of aldosterone receptors in the renal tubules of preterm infants leads to reduced sodium reabsorption in the final urine. During the first two to three days after birth, immature renal tubules in preterm infants cause sodium loss through the kidneys, leading to a negative sodium balance. Research by Gubhaju L and colleagues found that the fractional excretion of sodium in preterm infants' urine is negatively correlated with both gestational age and postnatal age. For preterm infants born at or before 28 weeks of gestation, the fractional excretion of sodium exceeded 6% on day 3 after birth, decreased to below 4% by day 7, and further declined to around 2% by one month of age (19). Excessive urinary sodium loss also results in the loss of body water. Although the kidneys of preterm infants exhibit a high filtration state, the overall glomerular filtration rate remains significantly low due to the absolute insufficiency in the total number of nephrons.

### 3.2 Low physiological requirements vs. High Therapeutic Demands

The majority of preterm infants require admission to the NICU for continuous monitoring and standardized management. Due to the immaturity of their digestive systems, oral feeding in preterm infants must be gradually increased to allow the underdeveloped gastrointestinal tract to adapt progressively to the extrauterine environment. In the early postnatal period, because the volume of oral feeding often does not meet their physiological needs, most preterm infants require supplemental energy through parenteral nutrition. Additionally, some preterm infants undergoing specialized treatments may receive fluids that include blood products, vasoactive medications, and agents promoting organ maturation, resulting in an overall higher fluid requirement.

Preterm infants experience a physiological phase of weight loss. After birth, neonates undergo a process of extracellular matrix fluid loss, which represents an adaptation to the extrauterine environment (20). Shortly after birth, the contraction of interstitial fluid is regulated by atrial natriuretic peptide and antidiuretic hormone (21). At approximately 8 weeks of gestational age, fetal water content constitutes about 94% of body weight; this proportion decreases with advancing gestational age, reaching approximately 86% at 24 weeks and 75% at full term. Concurrently, the distribution of body water shifts, with intracellular water content increasing with gestational age—reaching up to 34% at term—while extracellular water content exhibits an inverse trend (22, 23). Consequently, preterm infants born at earlier gestational ages possess higher total body water and extracellular fluid volumes. In full-term neonates, a weight loss of up to 10% within the first week postpartum is considered physiological; in preterm infants, this physiological weight loss may reach 15% and typically requires a longer recovery period. This physiological weight loss facilitates the elimination of excess body fluid, thereby reducing the risk of edema and FO. Excessive fluid administration during this period impedes the contraction of the interstitial space, leading to fluid accumulation extracellularly; if this exceeds the compensatory capacity of lymphatic return, FO ensues (24). Given the immature renal function and limited fluid handling capacity of preterm infants, coupled with the necessity to prevent complications across multiple organ systems, early physiological fluid requirements must be strictly controlled at low levels. This requirement often conflicts with the fluid demands associated with NICU management.

### 3.3 Dysfunction of the endothelial glycocalyx

The endothelial glycocalyx is a polysaccharide-rich layer produced by vascular endothelial cells. It primarily comprises glycoproteins, proteoglycans, and glycosaminoglycans. This glycocalyx is concentrated on the innermost surface of blood vessels and plays critical roles in cellular signal transduction, ion buffering, anti-inflammatory responses, antithrombotic functions, and mechanosensation-mechanotransduction (25). The endothelial glycocalyx contains five types of sulfated glycosaminoglycans, which are composed of long repeating disaccharide units forming glycosaminoglycan chains. These include heparan sulfate, chondroitin sulfate, keratan sulfate, dermatan sulfate, and hyaluronic acid. Among them, heparan sulfate is the most abundant, accounting for 50%–90% of all glycosaminoglycans attached to proteoglycans on the surface of vascular endothelial cells. These sulfated glycosaminoglycans give the glycocalyx an overall negative charge, which helps prevent cationic molecules from leaking out of the vascular system (26). This plays a crucial role in maintaining plasma osmotic pressure, reducing plasma transmembrane leakage, and preventing reabsorption into the surrounding interstitial space (27). Preterm infants are prone to infections, hypoxic-ischemic injury, ischemia-reperfusion damage, and rapid intravenous infusions, all of which can cause damage and shedding of the endothelial glycocalyx. Consequently, negatively charged sulfated glycosaminoglycans like heparan sulfate detach from the glycocalyx. Additionally, preterm infants experience various stresses after birth that increase the release of pro-inflammatory cytokines, which promote the activation of heparanase-1. This enzyme plays a fundamental role in the cleavage and transformation of heparan sulfate from glycocalyx proteoglycans, leading to glycocalyx degradation. These factors collectively result in glycocalyx damage, increased vascular permeability, plasma albumin leakage, and ultimately fluid retention (28).

### 3.4 Insufficient albumin synthesis

Preterm infants often exhibit immature hepatic function, resulting in a rapid turnover of the albumin pool and a slow synthesis rate. This leads to a reduction in plasma colloid osmotic pressure, causing excessive fluid accumulation within the interstitial spaces and subsequent edema. Inappropriate fluid administration can further exacerbate FO (29). Plasma osmotic pressure comprises both colloid osmotic pressure and crystalloid osmotic pressure, with colloid osmotic pressure playing a critical role in maintaining fluid balance between the intravascular and interstitial compartments. Various macromolecular proteins primarily sustain colloid osmotic pressure, among which albumin possesses a higher molar concentration compared to other plasma proteins, thereby accounting for the majority of colloid osmotic pressure maintenance (30). The effective filtration pressure, which drives plasma filtration from capillaries to form interstitial fluid, is defined by the equation: Effective Filtration Pressure = (Capillary Hydrostatic Pressure + Interstitial Fluid Colloid Osmotic Pressure) − (Interstitial Hydrostatic Pressure + Plasma Colloid Osmotic Pressure) (31). This relationship indicates that higher plasma albumin levels correspond to increased plasma colloid osmotic pressure and decreased effective filtration pressure, thereby promoting intravascular fluid retention and preventing tissue edema.

Currently, there is no universally established normal range for serum albumin levels in preterm infants; however, moderate hypoalbuminemia is commonly defined as plasma albumin concentration below 25 g/L, and severe hypoalbuminemia as below 20 g/L (8). In an intervention study focusing on extremely preterm infants, a randomized controlled trial involving 30 preterm infants with a mean gestational age of 29 weeks compared the short-term physiological responses between an albumin infusion group and a placebo group. The findings revealed contrasting trends in weight changes between the two groups: infants receiving albumin infusion experienced a significant weight loss (mean difference 95% confidence interval: −14 to −4.9 g, *P* < 0.01), whereas those in the placebo group showed a significant weight gain (mean difference 95% confidence interval: 0.3–82.5 g, *P* < 0.05) (32). The results indicate that preterm infants receiving albumin infusion exhibit a significant reduction in body weight, with the difference reaching statistical significance. This suggests that albumin infusion may effectively alleviate fluid overload in preterm infants by promoting fluid excretion. These results indirectly suggest that hypoalbuminemia may contribute to the development of FO.

## 4 The impact of early fluid overload on adverse outcomes in preterm infants

**Figure 1:**
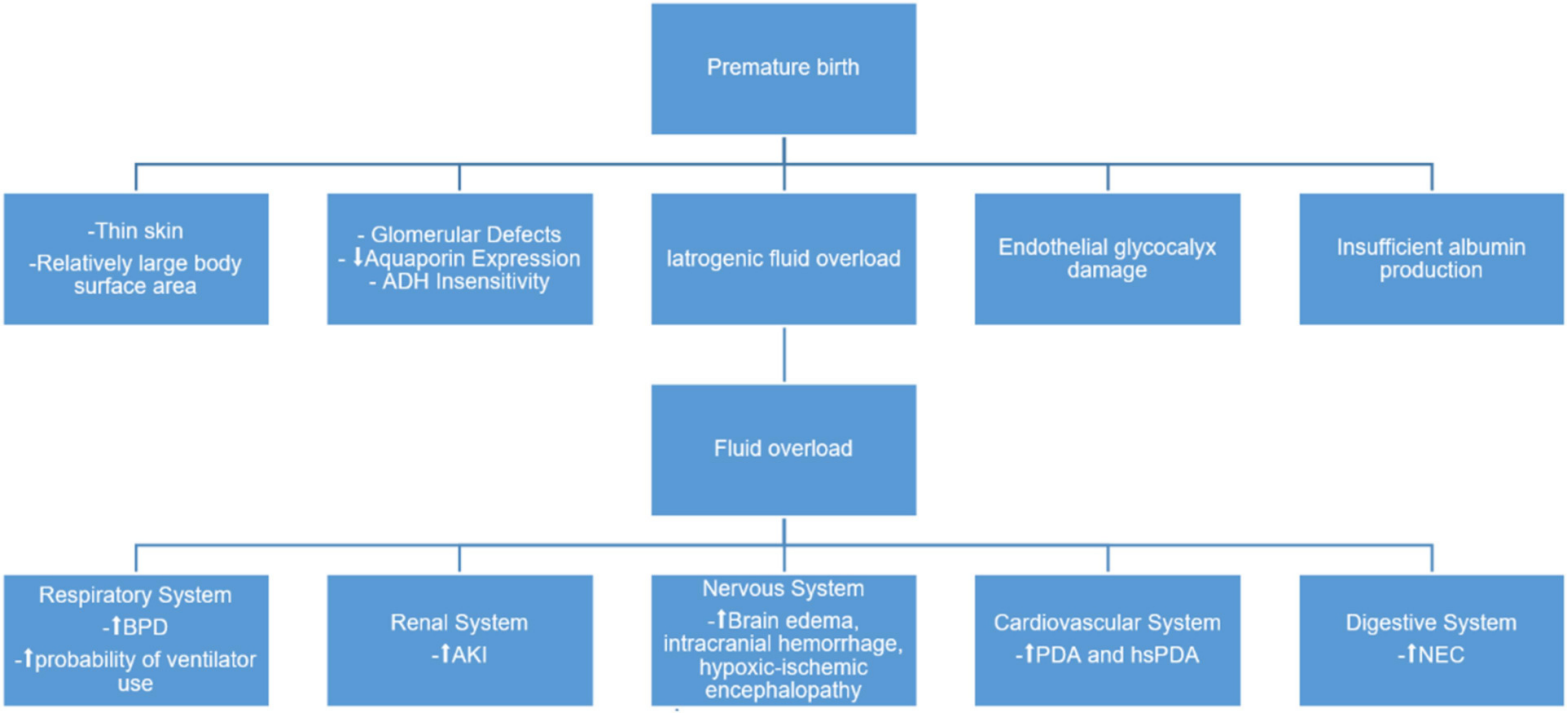
Fluid overload in preterm infants: pathophysiology and outcomes.

Although a direct causal relationship between FO and mortality in preterm infants has not yet been established, FO is known to adversely affect nearly all organ systems. For instance, it is closely associated with the development of bronchopulmonary dysplasia, pulmonary infections, patent ductus arteriosus, brain injury, and necrotizing enterocolitis in preterm infants. The occurrence of these systemic conditions demonstrates a significant correlation with the presence of FO. Figure 1 illustrates the pathophysiological mechanisms and outcomes associated with FO in preterm infants.

### 4.1 Respiratory system

Compared to full-term infants, preterm infants exhibit a reduced number of alveoli, underdeveloped pulmonary vasculature, and a diminished effective surface area for gas exchange. These vulnerabilities, compounded by factors such as mechanical ventilation, high-concentration oxygen therapy, prenatal infections and inflammation, patent ductus arteriosus, and postnatal infections, predispose preterm infants to bronchopulmonary dysplasia (BPD). BPD is characterized by impaired lung development, alveolar hypoplasia, and pulmonary vascular abnormalities (33). An increasing body of evidence indicates a strong association between FO and the incidence of BPD. For instance, a multivariate analysis study showed that preterm infants who develop BPD receive significantly higher daily fluid intake during the first five days post-birth compared to those without BPD after excluding factors such as gestational age and intrauterine infection (OR: 1.063, 95% CI: 1.006–1.123); although fluid intake remains elevated in the BPD group during days six to seven, this difference is not statistically significant (34). A cohort study involving 191 preterm infants born before 28 weeks of gestation identified a close correlation between FO within the first ten days of life and the development of BPD (OR: 1.60, 95% CI: 1.12–2.35) (35). Furthermore, AI-Jebawi et al. reported that infants with moderate to severe BPD had significantly higher fluid intake during the first week of life compared to controls, suggesting a relationship between fluid volume and BPD severity (36). Additionally, due to the deficiency of pulmonary surfactant and overall lung immaturity, the likelihood of requiring mechanical ventilation increases as gestational age decreases (37). Matsushita et al. found that in extremely preterm infants, FO within the first 72 h post-birth was directly associated with increased mortality. Their study also revealed that severe FO correlated with elevated mean airway pressure (MAP) on ventilator settings, indicating that greater fluid burden is linked to higher MAP values (38). A study utilizing data from the Assessment of Worldwide Acute Kidney Injury Epidemiology in Neonates (AWAKEN) database evaluated the impact of fluid balance during the first week of life in neonates diagnosed with acute kidney injury (AKI) (*n* = 645). The findings indicated that peak fluid balance and overall fluid status on day 7 post-birth were independently associated with the requirement for mechanical ventilation at the same time point. Compared to neonates who did not require mechanical ventilation, those who needed ventilatory support within the first seven days exhibited significantly higher peak fluid balance, lowest fluid balance, and fluid balance measurements on days 3 and 7 postnatally (39).

### 4.2 Renal system

Data from the AWAKEN database indicate that acute kidney injury (AKI) occurs in 29.9% of neonates during the early postnatal period. The incidence is notably higher among preterm infants born between 22 and 29 weeks of gestation, reaching 47.9%, compared to 18.3% in those born between 29 and 36 weeks, and 36.7% in preterm infants born at or beyond 36 weeks (40). In the ELGAN cohort, 38% of extremely preterm infants developed AKI, with nearly half of these cases being moderate to severe (18.2%). AKI can occur throughout the entire NICU hospitalization period, and the risk of occurrence is significantly negatively correlated with gestational age. The risk of severe AKI in infants born at 24 weeks gestation is twice and three times that of infants born at 26 and 27 weeks, respectively (41). A prospective cohort study investigating the association between FO and AKI in neonates included 58 term or near-term infants. Findings revealed that the AKI group (*n* = 9) exhibited a significantly greater percentage change in body weight by day 3 post-birth compared to the non-AKI group (*n* = 49), with median values of 8.2% (interquartile range: 4.4%–21.6%) vs. −4% (interquartile range: −6.5% to 0.0%), respectively (*P* < 0.001). The percentage change in weight on day 3 was calculated as [1 − (weight on day 3/birth weight)] × 100% (42). Accumulating evidence suggests that infants with AKI may experience higher fluid balance within the first seven days after birth. In summary, FO and AKI are independently and synergistically associated with adverse outcomes in critically ill preterm infants.

### 4.3 Cardiovascular system

The ductus arteriosus plays a crucial role in maintaining fetal circulation, typically undergoing functional closure within 1–2 days after birth in full-term neonates, whereas this closure is relatively delayed in preterm infants. The incidence of patent ductus arteriosus (PDA) is inversely correlated with gestational age: approximately 10% of neonates born between 30 and 37 weeks of gestation, 80% of those born between 25 and 28 weeks, and over 90% of neonates born before 24 weeks maintain an open ductus arteriosus at 4 days postnatally (43). While spontaneous closure of PDA is possible, persistent patency beyond a certain period necessitates pharmacological intervention. Hemodynamically significant patent ductus arteriosus (hsPDA) refers to cases in preterm or neonatal populations where PDA induces substantial hemodynamic alterations, resulting in increased cardiac workload and abnormal systemic blood flow distribution. Persistent hsPDA can lead to heart failure or hypotension, causing renal hypoperfusion and impaired fluid excretion, thereby exacerbating FO. Consequently, infants with hsPDA are more susceptible to FO compared to those with PDA alone. Rallis et al. investigated the relationship between FO on the first day of life and PDA in preterm infants born at or before 30 weeks of gestation, finding a significant association between FO ≥5% on day one and the occurrence of hsPDA (44). Earlier studies have also indicated that preterm infants with gestational age under 32 weeks and birth weight below 1,250 g who receive high fluid intake (>170 mL/kg/day) during the initial days post-birth exhibit an increased risk of PDA (45). Additionally, Depala et al. conducted a case-control study involving 108 infants diagnosed with PDA postnatally, demonstrating that increased fluid load correlates with a decreased likelihood of spontaneous PDA closure (46).

### 4.4 Nervous system

FO in preterm infants has been associated with an increased risk of cerebral edema, intracranial hemorrhage, and hypoxic-ischemic encephalopathy. Verma et al. investigated the relationship between early postnatal weight changes and the incidence of fluid metabolism-related complications in 101 preterm infants born before 29 weeks of gestation. Their findings indicated a protective threshold correlation whereby greater weight loss on day 5 was linked to a reduced risk of varying degrees of intraventricular hemorrhage (IVH) (47). Conversely, Aksoy et al. evaluated 126 extremely low birth weight infants and found a positive correlation between the extent of weight loss on day 3 and an increased incidence of IVH of any grade (48). Additionally, Zozaya et al. analyzed data from the Canadian Neonatal Network, examining the association between weight changes within the first three days after birth and mortality and/or severe neurological injury in 9,275 preterm infants (median gestational age = 26 weeks, interquartile range = 25–28 weeks). Their univariate analysis revealed a U-shaped relationship between weight change at day 3 and severe neurological injury, with the lowest risk observed within a weight change range of −13% to −9%. Weight loss exceeding this lower limit (< −13%) or weight gain above the upper limit (> −9%) was associated with an increased risk of neurological damage (49).

### 4.5 Digestive system

Necrotizing enterocolitis (NEC) is a severe intestinal disorder predominantly affecting neonates, particularly preterm infants. It is characterized by inflammation and necrosis of the intestinal tissue, which can lead to serious complications such as intestinal perforation and sepsis. Valentine et al. conducted a study involving 842 extremely preterm infants, examining the relationship between weight changes and fluid intake during the first week of life and subsequent clinical outcomes. Their findings indicated that maintaining a weight loss of 5% to 15% within the first seven days post-birth was associated with a significantly reduced risk of NEC (OR 0.49, 95% CI 0.25–0.98). Additionally, the study identified that a total fluid intake exceeding 150 mL/kg/day during this period correlated with an increased incidence of NEC (50). Furthermore, a Cochrane systematic review by Bell, which included four studies, demonstrated that fluid restriction in preterm infants significantly decreased the risk of NEC (24). Collectively, these findings underscore the critical role of early postnatal fluid management in preterm infants, suggesting that moderate regulation of fluid intake may contribute to lowering the risk of NEC development.

## 5 Early management strategies for FO in preterm infants

### 5.1 Precise fluid assessment

Accurate evaluation of fluid status in preterm infants is paramount for preventing FO and represents the most effective approach to its management. This involves meticulous monitoring of fluid intake and output, encompassing all sources of fluids such as parenteral nutrition and medication administration. Daily tracking of cumulative fluid balance in conjunction with relevant clinical parameters significantly mitigates the risk of FO progression. Given the rapid fluctuations in the internal environment of preterm infants, it is essential to promptly adjust fluid balance targets based on intravascular volume status. For extremely low birth weight infants with stable vital signs, fluid assessments are typically conducted every 12–24 h. In contrast, for those exhibiting unstable vital signs or abnormal fluid metabolism, the frequency of evaluation should be increased according to the infant's clinical condition (8). Assessment parameters include cumulative fluid intake and output over the specified period, daily body weight, clinical physical examination findings, and pertinent laboratory indices such as blood urea nitrogen, serum sodium, and blood pressure. Furthermore, accurate fluid assessment necessitates integration with appropriate auxiliary diagnostic tests, as outlined in the subsequent section.

#### 5.1.1 Noninvasive hemodynamic parameters

In preterm infants, bedside noninvasive cardiac function monitoring is commonly employed to assess hemodynamic alterations. Frequently utilized parameters include cardiac index (CI), cardiac output (CO), stroke volume (SV), stroke volume variation (SVV), pulse pressure variation (PPV), and peak aortic flow velocity (PAFV) (51). A study involving 120 preterm infants of varying gestational ages and birth weights measured noninvasive ultrasound-derived cardiac output parameters, revealing that SV, CO, CI, and myocardial contractility increased with advancing gestational age and birth weight, whereas heart rate decreased correspondingly. Additionally, corrected flow time (FTC) was observed to increase with gestational age (52). An international guideline suggests a reference range for CI in children of 3.5–5.5 L/(min m^2^) and for systemic vascular resistance index (SVRI) of 800–1,600 dyn·s·cm^−5^·m^2^ (53); however, values in neonates and preterm infants are typically lower than these ranges. A meta-analysis on pediatric fluid responsiveness indicated that the magnitude of respiratory variation in peak aortic flow velocity is the sole variable capable of predicting fluid responsiveness in children, whereas static parameters lack predictive value (54). In adult populations, PPV has been identified as the most specific and sensitive indicator of blood volume responsiveness. Compared to SVV, PPV appears more accurate in predicting intravascular volume status; however, its predictive utility is compromised by various confounding factors. Conditions such as intra-abdominal hypertension, arrhythmias, spontaneous breathing efforts, decreased chest wall compliance, and elevated respiratory rates relative to heart rate may all impair the accuracy of PPV assessment (55). Noninvasive cardiac function monitoring offers a safe and real-time evaluative tool for fluid management in preterm infants, yet its precision is constrained by technical, physiological, and equipment-related factors. Consequently, it should currently be regarded as a complementary modality to invasive monitoring rather than a complete substitute.

#### 5.1.2 Bioelectrical impedance analysis (BIA)

BIA involves the application of an alternating current to the body and the measurement of impedance or resistance changes associated with volume fluctuations. The underlying principle is that cardiac activity induces variations in thoracic blood volume, which in turn alter chest impedance. By placing electrodes on the neck and lower chest, these impedance changes can be recorded to calculate CO. BIA is also utilized to quantify total body water, intracellular fluid, extracellular fluid, protein, and fat levels, thereby facilitating the assessment of body fluid balance (56). Numerous studies have demonstrated that BIA provides reliable fluid status evaluation in dialysis patients (57, 58); however, its limitation lies in the inability to localize specific sites of extracellular volume expansion. Parameters derived from BIA regarding fluid distribution require adjustment for factors such as age, sex, and body composition. In adults, an overhydration to extracellular water ratio (OH/ECW) exceeding 15% indicates FO. In a cohort study involving healthy children aged two years and above, the observed OH/ECW was −1.0 ± 6.3% (59). Overhydration (OH) represents the difference between actual body weight and normohydrated body weight (calculated as lean tissue mass plus fat mass), and thus may be either positive or negative. Katherine et al. employed air displacement plethysmography (ADP) as the reference standard to compare the consistency between BIA and ADP in measuring fat-free mass in preterm infants. Their findings indicated that the agreement between BIA and ADP depended on the specific BIA equation used; the Dung, Lingwood, and Tint equations demonstrated the closest concordance with ADP, with mean biases (±95% confidence limits) ranging from 0.14 to 0.32 kg (60). Nonetheless, BIA measurements are influenced by numerous factors including body proportions, density, weight, length, resistivity coefficients, and the reference methods used to validate BIA accuracy. Even minor variations in any of these parameters can significantly impact the predictive outcomes (61). In preterm infants, rapid changes in the relative proportions of intracellular and extracellular water, coupled with their small body size, further complicate the estimation of fluid status via BIA. Consequently, standardized reference ranges for BIA-derived indices in this population are currently lacking, rendering assessment challenging. BIA is therefore more suitable for monitoring dynamic changes in hydration status over time. Given its noninvasive, convenient, rapid, and repeatable nature, future research aimed at developing more precise, preterm infant-specific BIA algorithms holds promise for enhancing its clinical utility.

#### 5.1.3 Echocardiography

Echocardiography offers numerous parameters for assessing volume overload, including left and right ventricular end-diastolic diameters, left ventricular end-systolic diameter, pre-ejection and ejection times of both ventricles, interventricular septal and left ventricular posterior wall motion range, as well as left atrial and aortic root dimensions. Both volume overload and acute pressure overload are associated with these measurable indices. Additionally, bedside stroke volume can be evaluated by measuring the velocity-time integral of the left ventricular outflow tract, facilitating the assessment of cardiac index and fluid responsiveness in individual patients, thereby helping to prevent iatrogenic FO (62). In preterm infants, ventricular diameters, vascular lumen sizes, and myocardial wall thicknesses show significant positive correlations with gestational age and birth weight (63). However, there is currently no internationally standardized threshold for functional echocardiographic diagnosis of FO in preterm infants of varying gestational ages. Previous studies involving 115 preterm infants compared right ventricular end-diastolic and end-systolic chamber areas and their percentage changes to those of term infants, establishing reference values stratified by birth weight (64). The inferior vena cava (IVC), a highly compliant vessel whose size varies with pressure changes, has been shown to correlate with fluid responsiveness (65). Noninvasive volume monitoring can be performed via ultrasound assessment of the IVC, with an IVC collapsibility index greater than 55% predicting fluid responsiveness (66). One study involving 32 mechanically ventilated children monitored respiratory variations in IVC diameter and peak aortic blood flow velocity within 10 min following a 10 mL/kg fluid bolus; results indicated moderate accuracy of IVC diameter variation in predicting fluid responsiveness in this population (area under the ROC curve = 0.70; optimal cutoff 7.7%, sensitivity 69.2%, specificity 78.9%, positive predictive value 69.2%, negative predictive value 78.9%) (67). Nonetheless, IVC diameter is influenced by multiple factors, including anatomical structure, respiration, and intra-abdominal pressure, necessitating integration with other diagnostic modalities (68). Given its noninvasive nature, cost-effectiveness, and ease of operation, echocardiography is widely utilized for evaluating FO in preterm infants.

#### 5.1.4 Chest radiography and ultrasound

Chest radiography remains the most commonly employed method for diagnosing acute FO. Key positive findings include central vascular congestion, interstitial edema with Kerley B lines, cardiomegaly, and pleural effusions (69). However, due to the absence of radiation exposure, lung ultrasound is increasingly favored in clinical practice. Lung ultrasound B-lines arise from widened interlobular septa caused by increased hydrostatic pressure or capillary permeability during pulmonary edema, allowing ultrasound wave propagation. The number of B-lines correlates with the severity of pulmonary edema and dynamically decreases following decongestive therapy (70). A meta-analysis focusing on adults with acute decompensated heart failure demonstrated that lung ultrasound exhibits superior sensitivity and specificity compared to chest radiography in diagnosing cardiogenic pulmonary edema (71). Marco Allinovi et al. conducted serial lung ultrasound assessments in 142 infants and children undergoing hemodialysis for end-stage renal disease or AKI, revealing a linear correlation between lung ultrasound B-line scores and FO estimated by body weight, thereby confirming the feasibility of quantifying FO via lung ultrasound (72). Additionally, bedside abdominal ultrasound can assist in diagnosing ascites, bowel wall edema, and intestinal obstruction (68), while jugular vein ultrasound aids in evaluating jugular venous pressure (73), both contributing to the auxiliary diagnosis of FO.

#### 5.1.5 Blood volume monitoring

Quantitative blood volume analysis (BVA) serves as an adjunctive tool for diagnosing FO by monitoring blood volume. This technique is based on indicator dilution methods, wherein a known quantity of a tracer is introduced into an unknown volume, and its concentration is measured to calculate the volume. Currently, radioactive iodine-labeled albumin (I-131) is commonly used as the tracer (74). In preterm infants, blood volume ranges from 85 to 110 mL/kg. Normal total blood volume (BV) is defined as a measured volume within ±8% of the expected normal value. Mild to moderate BV expansion is defined as an 8%–25% increase, while severe expansion is ≥25% above normal volume (75). Strobeck et al. retrospectively studied 245 patients with acute heart failure and demonstrated that BVA-guided volume management reduced the risk of mortality and rehospitalization due to acute heart failure (76). Although BVA can quantitatively diagnose FO in preterm infants, its application is limited by the need for radioactive tracers and steady-state conditions, rendering it less suitable for hemodynamically unstable infants or those undergoing acute volume shifts. Future development of safer tracers may enhance its applicability in this population. 4.1.6 Near-Infrared Spectroscopy (NIRS).

Near-Infrared Spectroscopy (NIRS) can be used to assess local tissue oxygenation and shows potential in detecting changes in end-organ perfusion caused by FO. NIRS is based on the scattering and absorption characteristics of biological tissues to near-infrared light (700–1,000 nm). In this wavelength range, oxygenated and deoxygenated hemoglobin have distinct absorption spectra, and tissues like bone allow good penetration. By placing a probe on the body surface that emits and receives reflected light, NIRS measures the mixed blood oxygen saturation (rSO_2_) of local tissues, providing real-time information on the oxygen supply-demand balance in the monitored area (77). Although there is currently no direct evidence linking NIRS parameters to the quantification of FO, heart failure and pulmonary edema caused by FO can lead to decreased local oxygen saturation (rSO_2_) in organs such as the brain and kidneys. In preterm infants, NIRS has been used to monitor oxygenation changes in the brain, kidneys, and intestines (78), Janaillac et al. conducted a study involving 20 preterm infants born at less than 28 weeks of gestational age and found that, under conditions of low cardiac output, cerebral regional tissue oxygen saturation and preductal perfusion index were strongly correlated with cardiac output (79). suggesting it may serve as an auxiliary tool to indirectly reflect physiological disturbances caused by FO. At present, empirical studies directly applying NIRS for FO monitoring are still limited, and the evidence base needs further development.

Given the unique physiological characteristics of preterm infants, FO assessment is more challenging than in adults and requires comprehensive evaluation integrating the aforementioned parameters. Fade et al. conducted a prospective cross-sectional study involving 60 children with chronic kidney disease undergoing hemodialysis, demonstrating that BIA, lung ultrasound, and IVC measurements have complementary effects in fluid assessment. When combined with clinical data, these modalities provide a more accurate evaluation of fluid status, with lung ultrasound B-line detection outperforming the other two methods in identifying subclinical FO (80). Comparison of monitoring methods is presented in Table 2.

**Table 2:** Comparison of invasive and Non-invasive tools for assessing FO in preterm infants.

Type	Assessment tool	Principle/parameters measured	Advantages	Limitations	Applicability in preterm infants
Invasive	Central Venous Pressure (CVP)	Measures pressure in the right atrium or superior vena cava, reflecting cardiac preload.	- Direct, continuous reading - Considered a gold-standard	- Highly invasive, significant risks (infection, pneumothorax, thrombosis) - Primarily reflects right heart preload; influenced by cardiac function & intrathoracic pressure - Accuracy limited in critically ill infants with variable vascular tone	Low.
	Quantitative blood volume analysis (BVA)	Directly measures total blood volume by analyzing the dilution curve of an intravenously injected tracer (I-131).	- Provides direct, quantitative blood volume data - Helps accurately differentiate absolute hypovolemia from relative hypovolemia due to vasodilation	- Invasive procedure requiring precise IV injection and blood sampling - Safety data of tracers in neonates requires consideration - Complex operation, high cost	Low.
Non-Invasive	Echocardiography	Uses ultrasound to assess cardiac structure and function, including LVEDD, LVESD, RVEDD, IVS, PWE, AOD, LAD, LVPEP, LVET, RVPEP, RVET, VTI, IVC	- Safe, repeatable - Provides integrated, real-time assessment of cardiac function and filling pressures	- Operator-dependent - Provides “snapshot” data, not continuous monitoring - Some parameters have limited utility in spontaneously breathing infants	High.
	Lung Ultrasound (LUS)	Detects ultrasound artifacts (“B-lines” or “white lung”) arising from fluid accumulation in lung tissues.	- Rapid, bedside availability - High sensitivity for pulmonary edema/fluid overload	- Cannot differentiate cardiogenic from non-cardiogenic pulmonary edema (requires correlation with echocardiography) - Findings can be confounded by underlying lung disease (e.g., RDS)	Very High.
	Bioelectrical Impedance Analysis (BIA)	Measures the body's resistance to a weak electrical current to analyze fluid distribution (quantify total body water, intracellular/extracellular fluid, protein, and fat levels).	- Simple to perform - Can quantitatively assess extracellular fluid levels; sensitive to fluid retention	- Reference values and interpretation criteria for preterm infants are still being refined - Unable to determine the exact location of extracellular volume expansion. - Rapidly changing ratios of intra- to extracellular water and the small body size of preterm infants specifically impair estimation accuracy.	Low. Emerging/Research.
	Noninvasive Hemodynamic Parameters	Uses technologies like thoracic electrical bioimpedance to non-invasively and continuously monitor CO, CI, SV, SVV, PPV, PAFV, etc.	- Provides continuous trend data - Response to preload change can assess fluid responsiveness - Relatively simple operation	- Susceptible to interference from patient movement, electrode placement, etc. - Provides relative trends; absolute values require cautious interpretation	Medium to High.

LVEDD, left ventricular end-diastolic diameter; LVESD, left ventricular end-systolic diameter; RVEDD, right ventricular end-diastolic diameter; IVS, interventricular septum thickness; PWE, posterior wall thickness; AOD, aortic root diameter; LAD, left atrial diameter; LVPEP, left ventricular pre-ejection period; LVET, left ventricular ejection time; RVPEP, right ventricular pre-ejection period; RVET, right ventricular ejection time; VTI, velocity time integral; IVC, inferior vena cava. CO, cardiac output; CI, cardiac index; SV, stroke volume; SVV, stroke volume variation; PPV, pulse pressure variation; PAFV, pulmonary artery flow velocity.

### 5.2 Appropriate fluid restriction

Early implementation of appropriate fluid restriction in preterm infants is associated with improved outcomes. A Cochrane systematic review evaluating four studies found that fluid restriction significantly reduced the risks of PDA, NEC, and mortality in preterm infants. There was also a nonsignificant trend toward reduced BPD risk (24). An investigation into early enteral feeding in extremely preterm infants revealed that early enteral nutrition reduced total fluid intake (enteral plus parenteral) during the first postnatal week and prevented excessive weight loss within the first 14 days. Moreover, increased enteral feeding volume during the initial 96 h post-birth may have long-term benefits on growth and cognitive outcomes (81). However, excessive fluid restriction may lead to severe dehydration, hypotension, reduced visceral perfusion, growth retardation, intracranial hemorrhage, and hyperbilirubinemia, necessitating cautious clinical evaluation of fluid restriction parameters.

### 5.3 Additional pharmacological interventions

Diuretics are employed to improve urine output in oliguric or anuric preterm infants. Furosemide is commonly used, with oral dosing in preterm infants at 1–2 mg/kg per dose every 12–24 h, not exceeding 6 mg/kg daily. Intravenous dosing ranges from 0.5 to 1.5 mg/kg per dose every 12–24 h. For preterm infants with glomerular filtration rates below 10 mL/min, continuous intravenous infusion of furosemide at 0.1–0.4 mg/kg/h is more effective (82). Studies have demonstrated that intraoperative urine output response to furosemide in preterm infants undergoing cardiopulmonary bypass predicts the development of cardiac surgery-associated AKI (83). Conversely, systematic reviews do not support routine diuretic use in preterm infants with neonatal respiratory distress syndrome (NRDS), as transient pulmonary function improvements induced by furosemide do not outweigh increased risks of PDA and hemodynamic instability (84). Bumetanide has also been shown to achieve negative fluid balance via continuous infusion in critically ill neonates, with oral dosing at 0.01–0.1 mg/kg per dose every 12–24 h, intravenous dosing at 0.01–0.05 mg/kg per dose every 12–24 h, and continuous intravenous infusion at 5–10 *μ*g/kg/h (85). Guidelines recommend maintaining urine output above 0.5–1 mL/kg/h in preterm infants receiving extracorporeal membrane oxygenation to prevent FO (86). While diuretics promote the excretion of electrolytes and water, they also carry potential side effects. Loop diuretics commonly used in preterm infants may cause ototoxicity, nephrocalcinosis hyperuricemia, hyperuricemia, and hyperglycemia. Regarding electrolyte imbalances, they can lead to hypovolemia, hyponatremia, hypokalemia, and hypercalciuria. Thiazide diuretics are prone to causing hyperglycemia, insulin resistance, hyperlipidemia, and hypersensitivity reactions. In terms of electrolyte disturbances, thiazides more commonly cause hyponatremia, hypokalemia, and hyperuricemia. Therefore, the use of diuretics in preterm infants should be approached with caution (87).

Hypoalbuminemia can contribute to fluid retention. The AWAKEN study analyzed data from 531 neonates and found that each 0.1 g/dL decrease in serum albumin was associated with a 12% increase in AKI risk, exacerbating FO progression (88). Appropriate albumin supplementation can increase colloid osmotic pressure, reducing interstitial fluid accumulation. Guidelines suggest albumin replacement therapy when serum albumin falls below 25 g/L to reduce FO risk; specifically, preterm infants with albumin <20 g/L should receive 2 g/kg within 4 h, while those with levels between 20 and 25 g/L should receive 1 g/kg within 4 h (8). Infusion of 20% albumin, which has higher oncotic pressure, is preferred. However, some scholars argue that the correlation between hypoalbuminemia and edema in preterm infants is weak due to their low reflection coefficient (indicating reduced capillary barrier function to macromolecules), resulting in net fluid movement across capillaries being independent of colloid osmotic pressure gradients (89). Consequently, evidence supporting albumin infusion in preterm infants remains insufficient.

For preterm infants with cardiac dysfunction, vasoactive agents such as dopamine, dobutamine, and epinephrine may be used to improve cardiac output. In infants born before 32 weeks gestation, maternal cortisol transfer via the placenta suppresses hypothalamic-pituitary-adrenal axis activity through negative feedback, leading to reduced adrenocorticotropic hormone secretion and adrenal stimulation. This physiological adrenal insufficiency suggests potential benefits of corticosteroid replacement therapy (90). For example, hydrocortisone has been shown to improve refractory hypotension and may enhance cardiovascular function in the context of FO (91). Recommended initial dosing for infants under 35 weeks gestation is 1–2 mg/kg, followed by 0.5–1 mg/kg every 8–12 h; for late preterm and term infants, dosing every 6–8 h is advised, with treatment duration adjusted based on cardiovascular response (90). A summary of the relevant intervention methods is presented in Table 3.

**Table 3:** Intervention studies for fluid management in preterm infants.

Intervention method	References and types	Population	N	Methodology	Conclusion
Fluid restriction	Bell et al. (24). Cochrane systematic review	Preterm infants (<37 weeks)	Included 5 studies	Based on 5 RCTs comparing high-volume vs. restricted water intake, with protocol variations in timing/duration.	Restricted water vs. high volume: Significantly increased weight loss; reduced PDA & NEC. Non-significant trends: reduced BPD, hemorrhage, death. increased risk of dehydration
	Kothapally et al. (96). Systematic Review and Meta-Analysis	Very Preterm infants (<32 weeks)	Included 18 studies: Case-control (7); Prospective cohort (2); Retrospective cohort (9)	Meta-analysis was conducted using Review Manager 5.4 employing a random-effects model. Both adjusted and unadjusted odds ratios (ORs) were combined using the inverse variance approach. Subgroup analyses were carried out based on study design and weight loss categories (excess weight loss greater than 15%; insufficient weight loss less than 5%). The degree of heterogeneity was measured using the I² statistic.	Excess (>15%) or inadequate (<5%) early postnatal weight loss in very preterm infants may be associated with higher odds of mortality, severe IVH, NEC, CLD
Pharmacological Interventions	Wright et al. (97). retrospective study	preterm infants ≤ 36 weeks	1,383	Evaluated the epidemiology of fluid balance in the first 14 postnatal days and its association with MV and mortality and studied the association of diuretics with fluid balance, MV, and mortality.	Diuretics show short-term benefits by increasing urine output (0.6 mL/kg/h) and reducing fluid balance. Although potentially lowering Day 7 mechanical ventilation risk in infants with 5%–15% fluid overload, this effect was not statistically significant. Importantly, diuretics failed to improve ultimate outcomes, warranting further investigation into their use in preterm infants with FO.
	Hagadorn et al. (98).	Not applicable.	Invite 600 neonatal specialists to participate in a survey questionnaire.	Designed 20 questions, including those about the conditions for using diuretics.	33% of experts choose to use diuretics when edema occurs in preterm infants, but most are more cautious due to side effects such as bone demineralization (83%), electrolyte imbalance (86%), and kidney calcification (81%).
	Jardine et al. (99). Cochrane systematic review	Preterm infants (<37 weeks)	Included 5 studies	Included: RCTs/quasi-RCTs (individual allocation). Comparison: Albumin infusion (single/repeated) vs. placebo/no treatment.	Fluid input versus output in 12 h periods preceding infusion, during infusion and post infusion. No significant differences were reported

IVH, intraventricular haemorrhage; CLD, chronic lung disease; NEC, necrotising enterocolitis; MV, mechanical ventilation.

### 5.4 Continuous renal replacement therapy (CRRT)

Continuous renal replacement therapy (CRRT) is a continuous and controlled method of removing fluids and solutes for patients with hemodynamic instability. It is considered for critically ill children with FO greater than 10% when diuretics fail to reverse or maintain fluid balance (53). Early initiation of CRRT in critically ill children with severe FO can improve survival rates (92). The effectiveness and delivery of CRRT largely depend on having a reliable vascular access. In preterm infants, ultrasound-guided catheterization of the left internal jugular vein via the supraclavicular or infraclavicular approach is the preferred choice due to the vessel's large diameter and resistance to collapse (93). Since preterm infants have relatively small total blood volume, transfusing red blood cells before CRRT to maintain adequate hemoglobin levels and sufficient circulatory perfusion, along with the use of vasoactive drugs to sustain stable blood pressure, helps ensure hemodynamic stability during CRRT (94). However, CRRT carries risks such as bleeding, thrombosis, and catheter dislodgement. Therefore, a thorough assessment of the child's vascular condition and coagulation function is essential before starting CRRT (95).

## 6 Ethical statement

All primary studies cited in this review have reported obtaining approval from institutional ethics committees as well as informed consent from the parents of the pediatric participants. As this study constitutes a secondary analysis of previously published literature and does not involve new patient data, it does not require additional ethical review.

## 7 Conclusion

**Figure 2:**
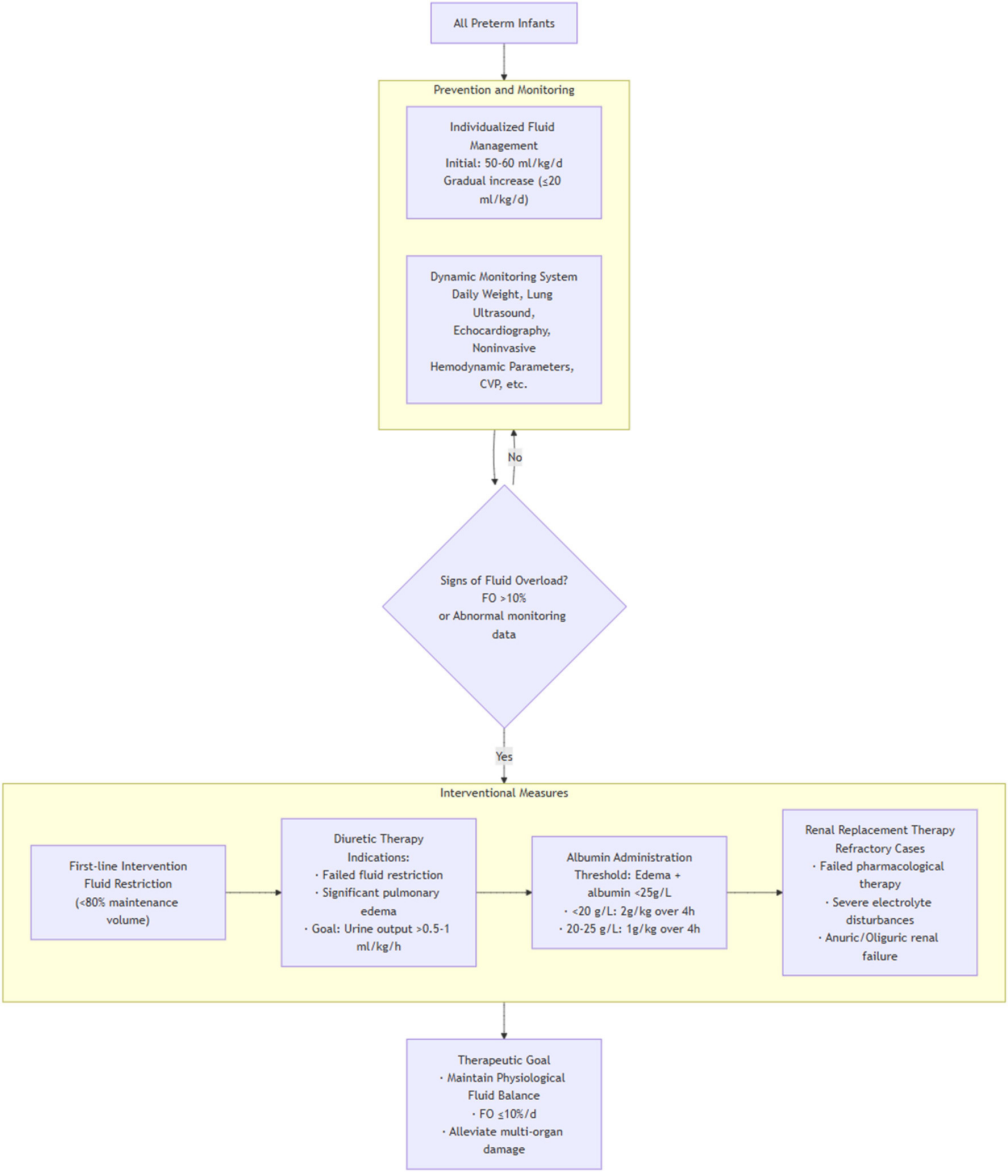
Clinical application framework.

FO in preterm infants is a complex process that adversely affects multiple organ systems and influences clinical outcomes. Comprehensive, multidimensional assessment of fluid balance combined with early targeted management of FO may improve prognosis. This review summarizes the mechanisms and advances in diagnosis and treatment of FO in preterm infants, aiming to enhance clinicians' understanding and promote cautious, standardized fluid therapy. despite providing important insights into the progress of research on FO in preterm infants, the existing evidence has significant limitations. Many studies are primarily based on observational research (such as the AWAKEN study), which carries a risk of residual confounding bias, and some controlled trials are few in number, have small sample sizes, and lack statistical power (for example, randomized controlled trials on albumin use and studies on the impact of FO on adverse outcomes in preterm infants). The definitions of FO and outcome measures vary across studies, further limiting the generalizability of the conclusions. Therefore, the management framework proposed in this review should be regarded as a practical guide based on the best available evidence rather than an absolute standard. Figure 2 presents a flowchart summarizing the prevention and intervention strategies for fluid overload in preterm infants.

Currently, there are still some challenges to overcome in the management of FO in preterm infants. First, the low body weight of preterm infants complicates fluid monitoring; while noninvasive techniques are safe and convenient, their accuracy requires improvement, whereas invasive methods offer greater precision but carry risks of injury and infection. Future integration of multiple clinical monitoring modalities with artificial intelligence algorithms may enable the development of multimodal assessment systems for more refined fluid management. Second, the unique physiology of preterm infants renders conventional drug dosing regimens potentially hazardous. Systematic investigation of precise dosing tailored to gestational age, weight, and pathological status is warranted to establish individualized pharmacotherapy strategies. Such approaches could facilitate targeted fluid management, reduce the incidence of FO, and improve outcomes in this vulnerable population.
